# Peptides Regulate Cortical Thymocytes Differentiation, Proliferation, and Apoptosis

**DOI:** 10.4061/2011/517137

**Published:** 2011-11-28

**Authors:** V. Kh. Khavinson, V. O. Polyakova, N. S. Linkova, A. V. Dudkov, I. M. Kvetnoy

**Affiliations:** ^1^Saint-Petersburg Institute of Bioregulation and Gerontology, RAMS pr. Dinamo 3, St. Petersburg 197110, Russia; ^2^I.P. Pavlov Institute of Physiology, RAS, Makarova emb. 6, St. Petersburg 199034, Russia

## Abstract

The processes of differentiation, proliferation, and apoptosis were studied in a cell culture of human cortical thymocytes under the influence of short peptides T-32 (Glu-Asp-Ala) and T-38 (Lys-Glu-Asp). Peptides T-32 and T-38 amplified cortical thymocytes differentiation towards regulatory T cells, increased their proliferative activity, and decreased the level of apoptosis. Moreover, peptides under study stimulated proliferative and antiapoptotic activity of the mature regulatory T cells.

## 1. Introduction

Being a major organ of the immune system, thymus plays a key role in the T cell immunity formation [1, 2]. An overt lowering of thymus functional activity in humans can be traced from the beginning of puberty and is associated with an early involution of this organ [3, 4]. Thymus involution is largely manifested by the reduced count of cortical thymocytes and mature T cells [1, 5]. Age-related reduction of thymus functional activity is a main cause of immunodeficiency, allergic reactions, respiratory infections, and other pathologies associated with a malfunction of the immune system [6, 7]. Therefore, the search of drugs able to revitalize thymus activity in its involution stage is one of the goals of immunology and gerontology [8–10]. The aim of the study has been to estimate the impact of the synthetic peptides on the differentiation of cortical thymocytes and mature T cells.

## 2. Materials and Methods

The cultures of human embryo thymus cells (14–26 weeks of gestation) were subject to investigation. They were studied in the following stages of their differentiation: immature cortical cells (CD4+CD8+) and mature regulatory T lymphocytes (CD4+CD25+).

Culture samples were obtained from the Laboratory of Cell Immunology of the Institute of the Federal Medical and Biological Agency. 4 control, and 12 experimental samples were incubated with synthetic peptides T-32 (Glu-Asp-Ala) and T-38 (Lys-Glu-Asp) at a single dose of 200 ng/mL for an hour at the temperature of 37°C. The above peptides were designed at the Saint-Petersburg Institute of Bioregulation and Gerontology of the Russian Academy of Medical Sciences (Russia). They were synthesized on the basis of amino acid sequences found in polypeptide extracts from the cattle thymus and vessels. It was previously determined that peptides T-32 and T-38 facilitated the expression of differentiation markers of thymes epithelial cells—proteins Pax1, Hoxa3, and TLP in cell culture. This effect was more pronounced in old cultures (8 passages) than in young cultures (1 passage) [11].Apart from that, the influence of peptides T-32 and T-38 on stem CD34^+^ cells of the bone marrow was investigated. Peptide T-38 stimulated stem cells expression of myeloid cells marker CD14 and marker of B-cells CD19 in the bone marrow, while peptide T-32 stimulated the expression marker CD19 only [11].

The functional activity of cells in the selected samples was studied in respect to the expression of the proapoptotic protein p53, antiapoptotic protein Mcl-1, and proliferation factor Ki-67. The same samples of the cell cultures underwent ultrastructural examination by means of transmitting electron microscopy and uranaffin reaction. Thymocyte cultures in the selected stages of their differentiation were prepared for further immunocytochemistry and electron microscopy studies in the following order.

1. Cell suspension was precipitated by centrifugation at 1000 rpm for 3 minutes. The resulting sediment in the quantity of 0,05–0,2 mL was set to resuspension in 3 mL of the 2,5% solution of the glutaralaldehyde on the 0,1 M of phosphate buffer (pH = 7,4). In 5 minutes the suspension of cells was centrifuged at 1000 rpm; the cells were rinsed with 0,1 M phosphate buffer for 2 minutes and then set to resuspension again in 6 mL of aldehyde fixative. After that, the content of each test was put into 2 tubes by 3 mL of suspension in each and fixed for 30 minutes.
2. The cells were rinsed with 0,1 M of the phosphate buffer 3 times for 10 minutes each time. At this stage, the samples for electron microscopy were extra-fixed in 1% osmium tetroxide for 45 minutes.
3. The cells for both studies were dehydrated by 70% ethanol (3 times for 10 minutes each), and those for electron microscopy were subject to additional resuspension in 2% *uranyl acetate for 12 hours. *
4. *The samples were subsequently dehydrated by 96% and 100% ethanol, then with 100% *acetone (3 times for 5 minutes each). Afterwards, the suspension of cells was soaked in the mixture of acetone and epon (1 : 1) for 1 hour.
5. One drop of the epon mixture was added to each plastic capsule. The sediment of cells in the amount of 1 mm³ was placed into these plastic capsules and left for 12 hours. Then the capsules were centrifuged at 1500 rpm for 10 minutes to achieve cell precipitation and then put into a thermostat at the temperature of 56°C for 48 hours. Semithin slices (1 *μ*m) and ultrathin slices (70 nm) were prepared on the ultra microtome LKB-7A (LKB, Sweden).

For the immunocytochemical study, semithin slices were stained with methylene blue-azure II main fuchsine. Afterwards, immunocytochemical method was undertaken with application of monoclonal antibodies to p53 (1 : 150), Mcl-1 (1 : 500), Ki-67 (1 : 250), and the sets for the visualization of the immunostaining (all the Novocastra reagents). Avidin-biotinylated horseradish peroxidase complex for visualization was employed with subsequent use of diaminobenzidine (ABS-kit, Dako).

An immunocytochemical study was carried out in strict accordance with a standard protocol.

Ultra-thin slices were used for electron microscopy. They were contrasted with *uranyl acetate and lead *citrate. The study was carried out on the electron microscope JEM-100S (JEOL, Japan). For ultrastructural analysis, whole cells and their fragments were photographed under the magnification of 14,000.

Verification of the secretory granules on the ultrastructural level was made using the uranaffin reaction. For this purpose cell sediment was fixed with 3% glutarate aldehyde on the 0,1 M cacodylic buffer (pH = 7,2) for 90 minutes. After that, 1 mm cut blocks were rinsed with 0,9% sodium chloride solution 3 times for 15 minutes each. The rinsed blocks were placed into 4% *uranyl acetate *water solution *(pH = 3,9) at 4 *°C* for 18 hours*. After the rinsing was done, the blocks were dehydrated in the alcohols and placed in epon.

An morphometric study was carried out using the computer analysis system of microscopic images, which consists of the Nikon Eclipse E400 microscope, digital camera Nikon DXM1200, and an Intel Pentium 4 computer with the “Morphology-Videotest 4.0” software installed on it. In each case, 10 fields of vision were analyzed at the magnification of 400. The surface of the expression was estimated by counting the proportion of immune-positive cells in the total area of the cells in the field of vision. The optical density of the expression was expressed in units. The optical density is a nondimensional value characterized by the level of light absorption. In our study, the optical density was estimated by Vidiotest-Morphology 5.0 to find out the ability of cells to express the investigated markers. The term “unit” in this paper is used to emphasize that the optical density is a nondimensional value and can be taken out if needed.

Cross-sectional expression of two molecules—markers of the thymocyte differentiation—was evaluated by a two-color flow cytometry method with application of monoclonal antibodies to CD8, marked with fluorescein thiocyanate *and the monoclonal antibodies to CD4, marked with phycoerytherin (Becton Dickinson, USA). In order to measure the expression of antibodies, the cells were incubated with the marked monoclonal antibodies at 37 *°C* for 30 minutes*. The binding of antibodies, marked with a fluorochrome, was monitored on the flow cytometry reader FACSCalibur (Becton Dickinson) with the help of the “CellQuest 3.1” software. In each case, 10,000 cells were analyzed in the rays of the argon laser (power—15 mWt, wavelength—448 nm) at the speed of 6,000 cells per second.

The results of the experiment were put out to the EXCEL (Microsoft) and STATISTICA 5.0 (Statsoft). For the purposes of measuring the mean, the Student's *t*-criterion with the double value of 95% was used.

## 3. Results of Investigation

Morphometric analysis has shown that the peptides under study revealed a stimulating effect on the proliferation of the immature cortical cells (CD4+CD8+) and differentiated regulatory T lymphocytes (CD4+CD25+).

**Figure 1:**
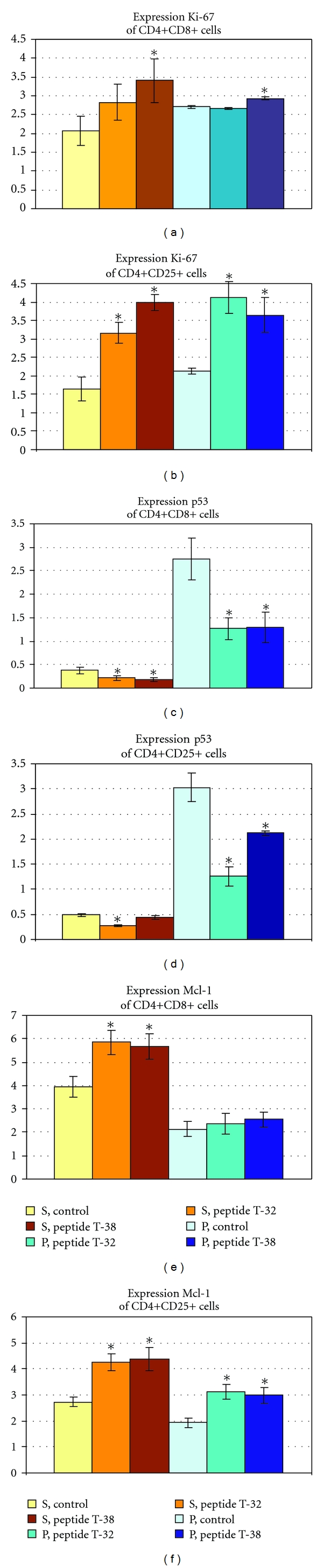
Expression of the marker of proliferation (Ki-67), apoptosis (p53) and antiapoptotic protein Mcl identified by immunocytochemical analysis in subpopulations of CD4+CD8+ and CD4+CD25+. *P < 0,05 as compared to the control group. S—area of the expression (%), P—optical density of the expression (units).

The area of the expression and the optical density of the proliferative marker Ki-67 on CD4+CD8+ membranes have risen reliably by 75% and 12% correspondingly under the influence of the peptide T-38, as compared to the control. Peptide T-32 did not show the same effect (Figure 1(a)).

At the same time, the effect of the peptides on the differentiation of CD4+CD25+ was more pronounced as compared to immature cortical cells. The area of the expression of the Ki-67 on the surface of the regulatory T lymphocytes under the action of both peptides under study has risen 2-2,3-fold, and the optical density was 1,7-2-fold in comparison with the control (Figure 1(b)).

Apart from that, peptides T-32 and T-38 prevented apoptosis of the differentiated and nondifferentiated thymocytes through the reduction of the expression of the proapoptotic protein p53 as well as through the increase in the level of the antiapoptotic protein Mcl.

The area of expression and the optical density of p53 have reduced approximately 2-fold under the influence of T-32 and T-38 on the CD4+CD8+ cells in comparison with the control (Figure 1(c)), whilst the area of the expression of the antiapoptotic marker Mcl has risen 1,4-1,5-fold after the incubation of the cortical thymocytes with the peptides. The peptides did not affect the optical density of the expression of Mcl CD4+CD8+ cells (Figure 1(c)).

Mature CD4+CD25+ cells appeared less sensitive to the antiapoptotic action of T-38 in comparison with the cortical thymocytes. So, the area of the expression of p53 did not change under the influence of T-38 and the optical density decreased only 1,3-fold (Figure 1(d)). When T-32 was added to the CD4+CD25+ cell culture, the area of the expression and the optical density of p53 reduced 2- and 2,2-fold, correspondingly in relation to the control (Figure 1(d)).

Both of the studied peptides contributed to the 1,5-fold increase in the area of the expression of the antiapoptotic factor Mcl, both in cortical thymocytes, and T-regulatory lymphocytes, as compared to the control (Figures 1(d) and 1(e)). However the optical density of the expression of the above-mentioned marker did not change reliably in the CD4+CD8+ cells under the influence of peptides T-32 and T-38, but did increase 1,3-fold in the differentiated T-regulatory cells (Figures 1(d) and 1(e)).

**Figure 2:**
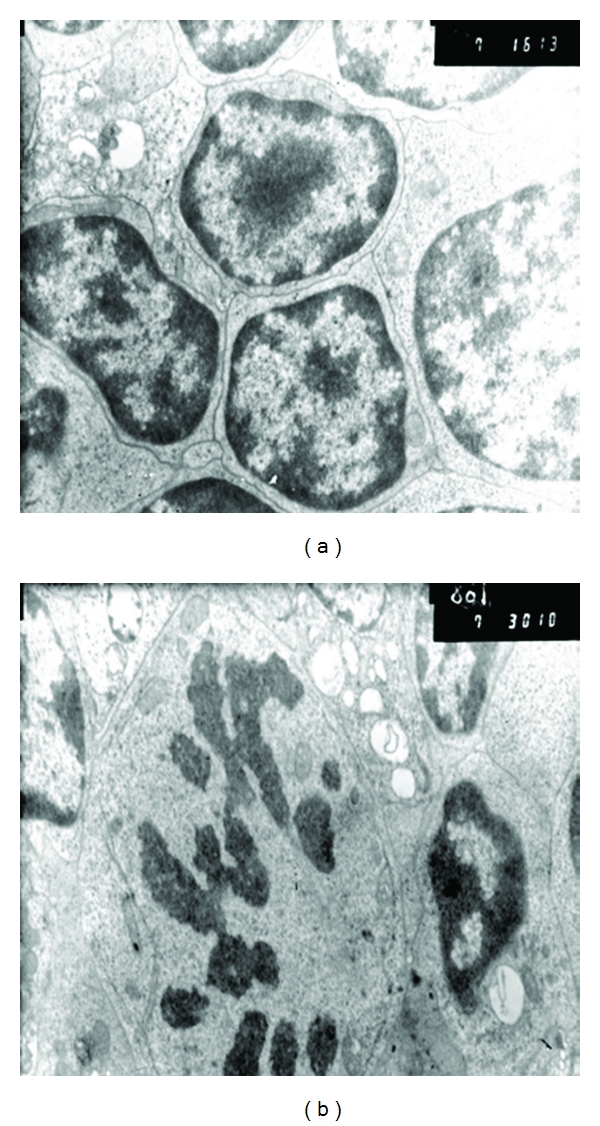
The effect of peptide T-38 on the proliferation of thymocytes, ×14,000: (a) proliferation of immature cortical cells (CD4+CD8+), (b) dividing regulatory T-lymphocyte (CD4+CD25+) in the metaphase stage.

The data obtained were confirmed by electron microscopy results. Peptides T-32 and T-38 promoted the increase in the quantity of mitosis figures amongst sliced and singularly placed cortical cells (Figure 2(a)). The ultrastructure of the cortical thymocytes was characterized by the weakly developed layer complex, and large quantity of the mitochondria and ribosomes localized close to the nuclei.

**Figure 3:**
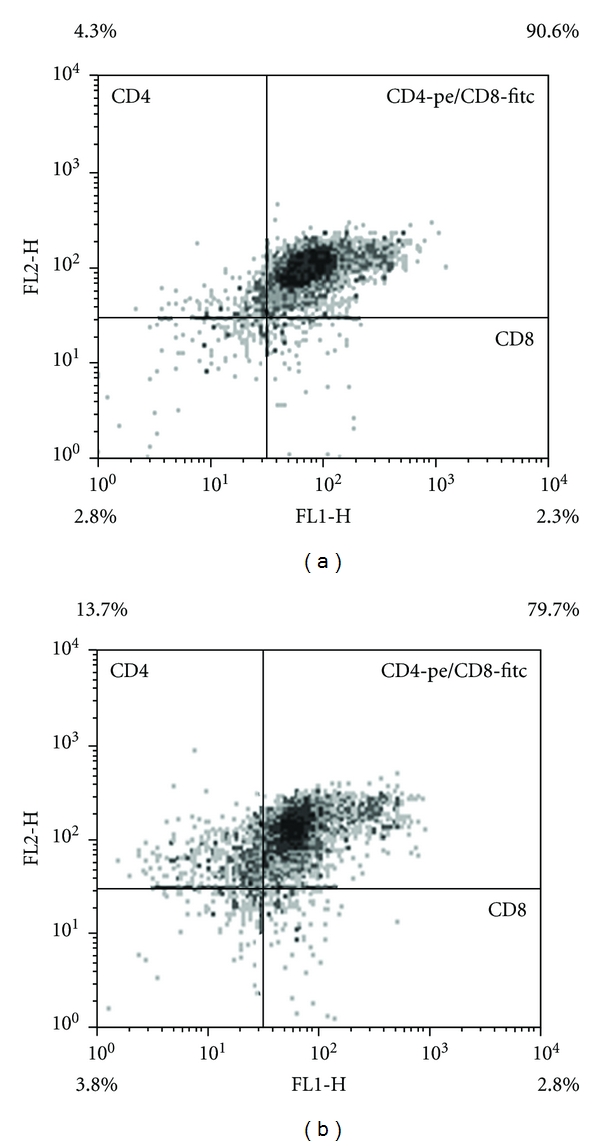
The proportion of CD4+CD8+ and CD4+CD8- thymocytes. (a) thymocytes control culture. (b) thymocytes culture with peptide T-38. CD4+ cells-two upper quadrants; CD8+ cells-two quadrants on the right.

A large quantity of cells which entered mitosis was registered under the influence of both peptides in the organotypic layers formed by the regulatory T lymphocytes (Figure 3(b)). Heterochromatin density increased in the nuclei of the regulatory T cells that are a part of the cells layers. Cytoplasm of the mature thymocytes manifested an increase in the quantity of endoplasmic reticulum and the complex layered elements following the administration of T-32 and T-38 peptides. This fact points at the intensified secretory functions of cells.

Indirect immunocytochemistry data which suggested possible T-32, and T-38—mediated stimulation of the cortical CD4+CD8+ thymocytes differentiation in the T-regulatory cells with phenotype CD4+CD8- was confirmed by the results of the flow cytometry. The quantity of CD4+CD8- cells made 4,3% in the control culture of the thymocytes with the total number of double positive CD4+CD8+ thymocytes—90,6%. The number of differentiated CD4+CD8- cells rose up to 13,7%, and the number of the double positive thymocytes went down to 79,7% under the effect of peptide T-38 (Figures 3(a) and 3(b)).

## 4. Discussion

Immunocytochemistry data evidence the ability of peptides T-32 and T-38 to induce differentiation of the cortical thymocytes in the direction of T-regulatory cells. The peptides facilitate proliferative activity, decrease the level of apoptosis, and increase the expression of antiapoptotic factors. The effect of peptide T-32 in its most is shown through its effect on the mature CD4+CD25+ cells. The action of T-38 was mostly targeted at the immature CD4+CD8+ thymocytes. Electron microscopy and cytometry studies confirmed the results received by the method of immunocytochemistry and pointed at the fact that synthetic peptides T-32 and T-38 are effective stimulators of differentiation and proliferation as well as inhibitors of thymocytes' apoptosis on different levels of their development.

The obtained results cohere with the experimental data which revealed a stimulating effect of the synthetic peptides of thymus and epiphysis on the differentiation of the polypotent cells [3, 6]. The retina peptides were shown earlier to stimulate proliferative activity of the pigmented epithelium [3]. The experimental results obtained and the literature data available suggest that the peptide regulation is based on the ability of peptides to stimulate the proliferative activity and differentiation of cells. It is worth mentioning that the given effects in different tissues are achieved only under the action of specific peptides.

## 5. Conclusion

The results of investigation evidence the property of peptide bioregulators to promote the enhanced functional activity of the thymus. Synthetic peptides reveal tissue-specific mechanism of action and targeted stimulation of cells, proliferative activity and differentiation.
